# Supplementary material for: Divergent Seasonal Reproductive Patterns in Syntopic Populations of Two Murine Species in Southern Spain, Mus spretus and Apodemus sylvaticus
Source: Animals (Basel). 2021 Jan 20;11(2):243. doi: 10.3390/ani11020243 (PMC7908971; doi:10.3390/ani11020243)
Supplement: Supplementary file 1 [file animals-11-00243-s001.zip › Figure_S1.pdf]

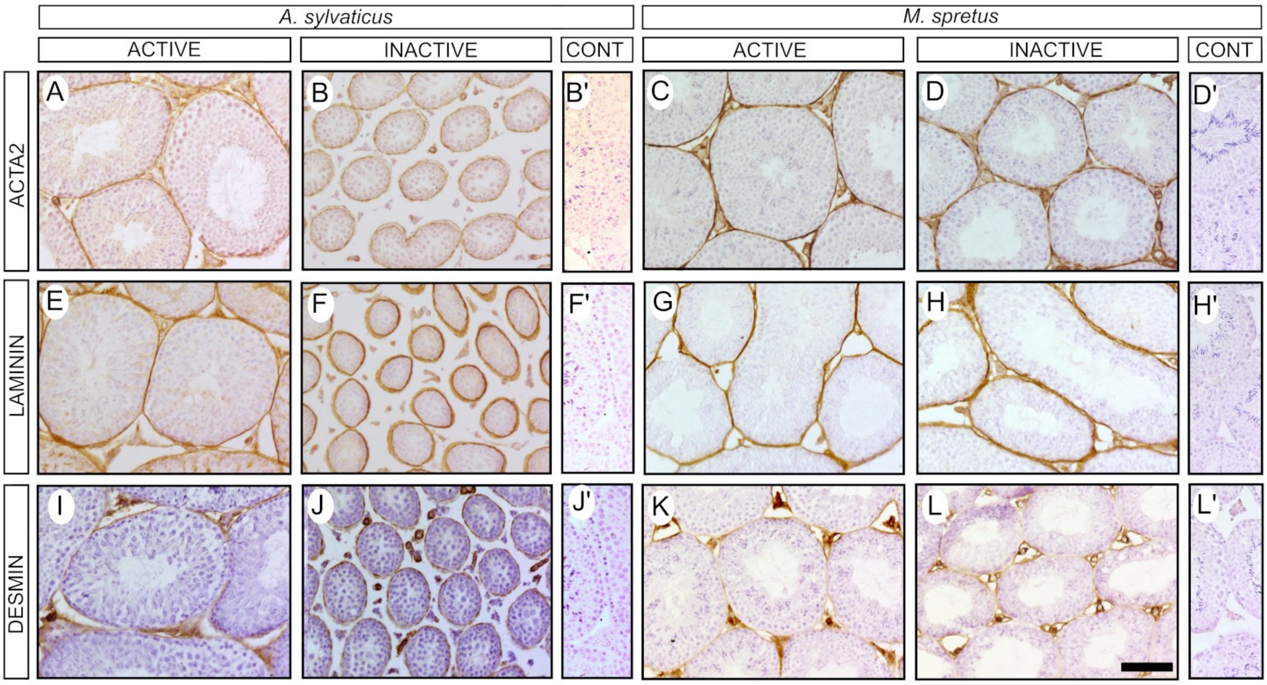

Figure S1. Immunohistochemistry for several molecular markers of the *lamina propria* in testis sections of *A. sylvaticus* and *M. spretus*. Immunohistochemistry for ACTA2 (A-D), LAMININ (E-H), and DESMIN (I-L) in active (A,E,I) and inactive (B,F,J) testes of *A. sylvaticus* and in active (C,G,K) and inactive (D,H,L) testes of *M. spretus*. Inserts at B′,D′,F′,H′,J′,L′ show negative controls. Scale bar shown in L represents 100 μm for all the pictures.
